# Molecular detection and genetic characterisation of pathogenic *Theileria, Anaplasma* and *Ehrlichia* species among apparently healthy sheep in central and western Kenya

**DOI:** 10.4102/ojvr.v86i1.1630

**Published:** 2019-06-13

**Authors:** Aaron E. Ringo, Gabriel O. Aboge, Paul F. Adjou Moumouni, Seung Hun Lee, Charoonluk Jirapattharasate, Mingming Liu, Yang Gao, Huanping Guo, Weiqing Zheng, Artemis Efstratiou, Eloiza M. Galon, Jixu Li, Oriel Thekisoe, Noboru Inoue, Hiroshi Suzuki, Xuenan Xuan

**Affiliations:** 1 National Research Center for Protozoan Diseases, Obihiro University of Agriculture and Veterinary Medicine, Obihiro, Japan; 2 Department of Public Health, Pharmacology and Toxicology, Faculty of Veterinary Medicine, University of Nairobi, Nairobi, Kenya; 3 Department of Pre-clinic and Applied Animal Science, Faculty of Veterinary Science, Mahidol University, Phutthamonthon Nakhonpathom, Thailand; 4 Department of Disinfection and Vector Control, Nanchang Center for Disease Control and Prevention, Nanchang, China; 5 Unit for Environmental Sciences and Management, North-West University, Potchefstroom, South Africa; 6 Obihiro University of Agriculture and Veterinary Medicine, Obihiro, Japan

**Keywords:** sheep, PCR, Kenya, tick-borne pathogens, phylogenetic analysis

## Abstract

Tick-borne diseases (TBDs) caused by *Theileria, Babesia, Anaplasma* and *Ehrlichia* species are common in tropical and subtropical regions. In this study, we investigated the presence and genetic diversity of *Theileria* spp., *Anaplasma ovis, B. ovis, E. ruminantium* and *Anaplasma* spp. in sheep from the Machakos and Homa Bay counties of Kenya. In order to improve the diagnosis and control of ovine TBDs, a total of 76 blood samples from apparently healthy sheep were screened using a polymerase chain reaction (PCR). The assays were conducted using primers based on *Theileria* spp. *18S rRNA, Anaplasma ovis* Major surface protein-4 (*AoMSP4*), *B. ovis 18S rRNA, E. ruminantium pCS20* and *Anaplasma* spp. *16S rRNA*. The overall infection rates for *Theileria* spp., *A. ovis, E. ruminantium* and *Anaplasma* spp. were 39/76 (51.3%), 26/76 (34.2%), 6/76 (7.9%) and 31/76 (40.8%), respectively. The overall co-infection was 47/76 (61.8%). All *Theileria* spp. positive samples were confirmed to be of *Theileria ovis* on sequencing. A phylogenetic analysis of the *18S rRNA* gene sequences of *T. ovis* revealed that all isolates of this study clustered with *T. ovis* sequences extracted from the GenBank suggesting this gene is highly conserved. *E. ruminantium pCS20* sequences were in the same clade on the phylogenetic tree. However, three *AoMSP4* sequences from this study appeared in the same clade, while one sequence formed a separate branch revealing genetic divergence. The *16S rRNA* sequencing revealed uncharacterised *Anaplasma* spp. and *A. ovis*. The phylogenetic analyses of the uncharacterised *Anaplasma* spp. revealed that the two sequences from this study appear in an independent clade from other sequences extracted from the GenBank. This study provides important information regarding the occurrence of tick-borne pathogens and their degree of genetic diversity among sheep in Kenya, which is useful for the diagnosis and control of TBDs.

## Introduction

Tick-borne diseases (TBDs) caused by *Theileria, Babesia, Anaplasma* and *Ehrlichia* are common in tropical and subtropical regions of the world where there is increased interaction of hosts, pathogens and vectors (Bilgic et al. 2017). Over the years, limited attention of TBD studies has been directed to sheep and goats compared to cattle (Yin et al. 2007). However, small ruminants are becoming important in a number of countries as far as socio-economic importance is concerned. Therefore, more attention is now being directed towards pathogens of sheep and goats (Bilgic et al. 2017).

The tick-borne pathogens of small ruminants include *Theileria ovis, T. separata, T. lestoquardi, T.* sp. OT1, *T.* sp. OT3, *T. luwenshuni* and *T. recondita* which cause theileriosis. *Babesia ovis* and *B. motasi* cause babesiosis. *Anaplasma ovis* causes anaplasmosis, whereas *A. phagocytophilum* causes tick-borne fever and *Ehrlichia ruminantium* causes ehrlichiosis (Bilgic et al. 2017). *T. lestoquardi, B. ovis, B. motasi, A. phagocytophilum* and *E. ruminantium* are considered to be pathogenic, while the rest with the exception of the recently detected *T*. sp. OT1 and *T*. sp. OT3 are non-pathogenic in sheep and goats (Razmi & Yaghfoori 2003; Schnittger et al. 2000; Uilenberg 1995). The pathogenicity of *T*. sp. OT1 and *T*. sp. OT3 do not have conclusive evidence (Yin et al. 2007).

Economic losses incurred from the TBDs include mortality, production losses, veterinary costs and tick control (Jonsson, Bock & Jorgensen 2008). In sheep, *T. ovis* and *A. ovis* manifest themselves as sub-clinical infections (Bilgic et al. 2017). Animals that survive the acute phase of infection develop a life-long carrier state, which is associated with significant production and economic losses (Gharbi et al. 2006; Uilenberg 1995). Therefore, the pathogens regarded as less pathogenic should equally be considered important, as they continuously infect ticks when they are in the carrier state, resulting in new infection to uninfected animals (Razmi & Yaghfoori 2003).

The eastern part of Africa is one of the three regions of sub-Saharan Africa which are considered to be highly populated by sheep and other livestock (Hanotte et al. 2000). Regardless of the large population of sheep in the region, little information is available on the presence and genetic diversity of tick-borne pathogens. This study was conducted to fill in this information gap. Blood samples were collected from sheep in Machakos and Homa Bay counties in Kenya and tested for the occurrence and genetic diversity of ovine tick-borne pathogens using PCR and sequencing.

## Materials and methods

### Study area

**FIGURE 1:**
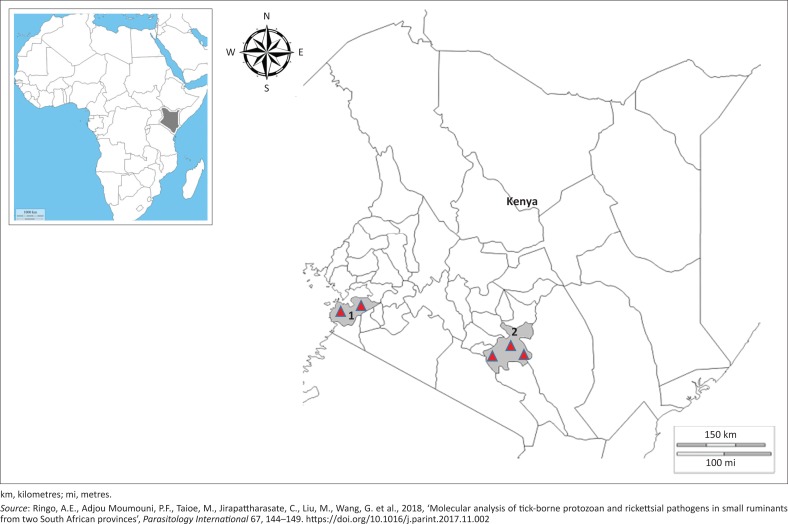
A map of Kenya showing the two counties where samples were collected: (1) Homa Bay in western Kenya and (2) Machakos in central Kenya. The red triangles are locations of sample collection farms.

Blood samples were collected from sheep in Machakos and Homa Bay counties (Figure 1). Machakos is located in central Kenya, about 63 km south-east of Nairobi. The local climate is semi-arid with hilly terrain and an altitude of 1000 m – 2100 m above sea level. The climate of this area is temperate and subtropical whereby temperatures are modified by altitude. The area has summer rainfall, while the vegetation is savannah, and rain starts at the end of March to May.

Homa Bay county is located in western Kenya along Lake Victoria. The county is near mount Homa and Ruma National Park, located 420 km south-west of Nairobi. The climate is semi-arid with temperatures ranging from 26 °C in the coldest months (April and November) to 34 °C during the hottest months (January and March). The county receives an average rainfall of 1100 mm, long rains in March to May and short rains in September to November (Climate-data.org 2018).

The farmers in both counties are mainly pastoralists with large groups of livestock involving cattle, sheep and goats. The breeds of sheep are mainly Red Maasai.

### Sample collection and DNA extraction

A total of 76 blood samples were collected in August 2011 (dry season) from clinically healthy sheep, using sterile needles and EDTA-coated vacutainer tubes (Boenmed, Jiangsu, China). An average of 3 mL – 5 mL of blood was drawn from the jugular vein of the animal and refrigerated until transported to the laboratory. Fifty-two and 24 samples were collected in Machakos and Homa Bay counties, respectively. Samples were collected randomly in five locations and mostly from pastoralist farms. Male and female sheep of around 2 years of age and above were targeted. DNA was extracted from 200 *µ*L of blood using the QIAamp DNA Blood Mini Kit (Qiagen, Hilden, Germany), following manufacturer’s protocol, and stored at -30 °C until the time of use.

### Molecular detection of tick-borne pathogens

All samples were screened using PCR with primers obtained from previous studies for *Theileria* spp. *18S rRNA, B. ovis 18S rRNA, A. ovis Major surface protein 4* (*AoMSP4*), *Anaplasma* spp. *16S rRNA* and *E. ruminantium pCS20* genes (Table 1). The reaction mixture had a final volume of 10 *µ*L, containing 0.5 mM of each primer, 1 *µ*L of 10×standard *Ex taq* buffer, 1 *µ*L of dNTP mix and 0.1 *µ*L of Ex taq polymerase (Takara – Shiga, Japan), 1 *µ*L of DNA template and 5.9 *µ*L of double distilled water. The double distilled water was used as a negative control, while positive controls were positive samples from a previous study (Ringo et al. 2018). Polymerase chain reactions were run in a thermal cycler (Bio Rad, Hercules, CA, United States [US]). Polymerase chain reaction conditions consisted of initial denaturation at 95°C for 5 minutes, followed by 35 cycles of denaturation for 1 min at 94 °C, 1 min annealing at differing temperatures which can be found in Table 1 and 1.30 min extension at 72 °C. The final extension was set to 10 min at 72 °C. The same PCR conditions were used for *Anaplasma* spp. PCR and nested PCR. As for *E. ruminantium*, the reaction mixture was performed in a semi-nested PCR consisting of initial denaturation for 3 min at 94 °C, followed by 25 cycles of denaturation for 0.30 seconds at 94 °C, annealing for 0.45 seconds at 61 °C and 1 min at 72 °C extension. The final extension was done at 72 °C for 10 min. The PCR products were electrophoresed on a 2% agarose gel and then stained with ethidium bromide and viewed under UV transilluminater.

**TABLE 1:** List of primers used for polymerase chain reaction assays.

Target gene	Assay	Primer sequences (5′ → 3′)		Fragment (bp)	Annealing temp (°C)	Reference
		Forward	Reverse			
*Babesia ovis* (*18S rRNA*)	PCR	TGGGCAGGACCTTGGTTCTTCT	CCGCGTAGCGCCGGCTAAATA	549	62	(Aktas, Altay & Dumanli 2007)
*Anaplasma ovis* (*AoMSP4*)	PCR	TGAAGGGAGCGGGGTCATGGG	GAGTAATTGCAGCCAGGCACTCT	347	62	(Alessandra & Santo 2012)
*Anaplasma* spp.(*16S rRNA*)	PCR	GGTTTAATTCGATGCAACGCGA	CGTATTCACCGTGGCATG	430	78–69	(Bekker et al. 2002)
	nPCR	GGTTTAATTCGATGCAACGCGA	GCTCAGCCTTGCGACGT	335	78–69	(Simuunza et al. 2010)
*Ehrlichia ruminantium* (*pCS20*)	PCR	ACTAGTAGAAATTGCACAATCYAT	RCTDGCWGCTTTYTGTTCAGCTAK	400	61	(Farougou et al. 2012)
	nPCR	ACTAGTAGAAATTGCACAATCYAT	TGATAACTTGGWGCRRGDARTCCTT	278	61	-
*Theileria* spp. (*18S rRNA*)	PCR	GAAACGGCTACCACATCT	AGTTTCCCCGTGTTGAGT	778	55	Cao et al. 2013
	nPCR	TTAAACCTCTTCCAGAGT	TCAGCCTTGCGACCATAC	581	55	-

PCR, polymerase chain reaction; nPCR, nested Polymerase Chain Reaction; bp, base pair.

### Cloning and sequencing

For sequencing, 3–5 positive samples per detected pathogen were randomly selected. Amplicons were purified using a QIAquick Gel Extraction Kit (Qiagen, Germany) per the manufacturer’s protocol. The concentration of the extracts was checked using the Nano Drop 2000 spectrophotometer. The template (6 *µ*L) was ligated into a pGEM-T Easy vector (2 *µ*L) (Promega, US), with T4 DNA ligase and restriction buffer (each 1 *µ*L) added and incubated at 16 °C for 3 hours and then at 4 °C overnight. Transformation of the plasmid into *Escherichia coli* DH5α competent cells (prepared in-house) was performed. Lysogeny broth (LB) was added and incubated at 37 °C in a shaker incubator for 1 h and then inoculated on LB agar plates and incubated at 37 °C overnight. Colonies were picked and put in LB broth with an antibiotic (Ampicillin) 50 *µ*g/mL (Wako, Saitama, Japan) incubated at 37 °C overnight in a shaker incubator. Plasmid was extracted using the NucleoSpin^®^ Plasmid QuickPure (Macherey-Nagel-Germany) kit. The samples were sequenced using a BigDye Terminator v3.1 Cycle Sequencing Kit (Applied Biosystems, US) and a 3100 Genetic Analyzer (Applied Biosystems, Foster city, Calafornia, US). The alignment of the sequences was performed using Lasergene v14.1 (DNASTAR, Madison, WI, US). The nucleotide sequence identities and similarities were determined by using a GenBank BLASTn analysis.

### Phylogenetic analysis

The sequences obtained in this study were compared with sequences deposited in the GenBank by phylogenetic analysis using MEGA version 7.0 software (Kumar, Stecher & Tamura 2016). The maximum likelihood method was used to construct phylogenetic trees for *T. ovis, A. ovis, E. ruminantium* and uncharacterised *Anaplasma* spp. Bootstrap analysis with 1000 replications was used to estimate the confidence of the nodes and branches of the trees.

### Nucleotide sequence accession numbers

Sequences obtained in this study were deposited in the GenBank database of the National Center for Biotechnology Information using BankIt. The sequences were assigned the following accession numbers: MF360021 to MF360025 for *T. ovis 18S rRNA*; MF360026 to MF360029 for *A. ovis MSP4*; MG637125 to MG637127 for uncharacterised *Anaplasma* spp. *16S rRNA*; and MG544303 to MG544305 for *E. ruminantium pCS20.*

### Ethical considerations

The owners of the selected farms were informed about the study and provided their approval for sample collection from their sheep. All the procedures were approved and carried out according to ethical guidelines for the use of animal samples permitted by Obihiro University of Agriculture and Veterinary Medicine (permit for animal experiment: 280080, DNA experiment 1219-2; Pathogen: 2015727).

## Results

### Overall infection rates

Polymerase chain reaction results revealed that 59/76 (77.6%) sheep were infected with at least one pathogen. Seventeen sheep (22.4%) were not infected with any of the screened pathogens. *Theileria* spp. had an overall infection rate of 39/76 (51.3%), *A. ovis* 26/76 (34.2%), *E. ruminantium* 6/76 (7.9%) and uncharacterised *Anaplasma* spp. 31/76 (40.8%). *Theileria ovis* was the only *Theileria* species identified following the sequencing of all PCR-positive *Theileria* spp., while for *Anaplasma* spp., the uncharacterised *Anaplasma* spp. and *A. ovis* were identified. Meanwhile, *B. ovis* and *T. lestoquardi* were not detected in this study.

### Infection rates based on location

In Homa Bay, 20/24 (83.3%) sheep were infected with at least one pathogen, while 4/24 (16.7%) were not infected with any of the pathogens screened. The infection rates for each pathogen in Homa Bay were 13/24 (54.2%), 8/24 (33.3%), 2/24 (8.3%) and 10/24 (41.7%) for *T. ovis, A. ovis, E. ruminantium* and uncharacterised *Anaplasma* spp., respectively. In Machakos, 39/52 (75%) sheep were infected with at least one pathogen. Thirteen of the 52 (25%) sheep were not infected with any of the screened pathogens. The infection rates for each pathogen in Machakos were 26/52 (50%), 18/52 (34.6%), 4/52 (7.7%) and 14/52 (26.9%) for *T. ovis, A. ovis, E. ruminantium* and uncharacterised *Anaplasma* spp., respectively.

### Mixed infections

Several sets of co-infections were revealed in this study (Table 2). Multiple infections were found in 47/76 (61.8%) samples. *Theileria ovis* was the pathogen most frequently associated with multiple infections (35/76; 46.1%) followed closely by *A. ovis* (34/76; 44.7%) (Table 2). *Theileria ovis* + *A. ovis* co-infection had the highest overall prevalence. A triple infection (*A. ovis* + *T. ovis* + uncharacterised *Anaplasma* spp.) with an overall prevalence of 6/76 (7.9%) was revealed in this study (Table 2).

**TABLE 2:** Mixed infections detected in sheep in this study.

Pathogens	County											
	Homa Bay (*n* = 24)				Machakos (*n* = 52)				Overall (*n* = 76)			
	Infected		Uninfected		Infected		Uninfected		Infected		Uninfected	
	*N*	%	*N*	%	*N*	%	*N*	%	*N*	%	*N*	%
*Theileria ovis* + *Anaplasma ovis*	4	16.7	20	83.3	14	26.9	38	73.1	18	23.7	58	76.3
*Anaplasma ovis* + uncharacterised *Anaplasma* spp.	3	12.5	21	87.5	5	9.6	47	90.4	8	10.5	68	89.5
*Anaplasma ovis* + *Ehrlichia ruminantium*	2	8.3	22	91.7	0	0.0	52	100.0	2	2.6	74	97.4
*Theileria ovis* + uncharacterised *Anaplasma* spp.	5	20.8	19	79.2	6	11.5	46	88.5	11	14.5	65	85.5
Uncharacterised *Anaplasma* spp. + *Ehrlichia ruminantium*	2	8.3	22	91.7	0	0.0	52	100.0	2	2.6	74	97.4
*Anaplasma ovis* + *Theileria ovis* + uncharacterised *Anaplasma* spp.	2	8.3	22	91.7	4	7.7	48	92.3	6	7.9	70	92.1

### Comparative sequence analysis

The PCR products of all the *T. ovis, A. ovis* and *E. ruminantium* isolated in this study were of the expected sizes of 520 bp (base pair), 347 bp and 279 bp, respectively. The three uncharacterised *Anaplasma* spp. amplified products were of different sizes (398 bp, 367 bp and 344 bp). The percentage of identity among the five *T. ovis 18S rRNA* nucleotide sequences of this study (MF360021 – MF360025) ranged from 99.61% to 100.00%. These sequences shared 100% identity with previous sequences deposited in GenBank from Sudan (AY260171 and MG333457) and Tanzania (AY260174). Furthermore, the four *A. ovis MSP4* nucleotide sequences of this study (MF360026 – MF360029) shared 97.98% – 100.00% nucleotide identity and had 98% nucleotide identity with the other sequences from Tunisia (KY659324, KY659320, KM285220 and KC432643). Meanwhile, the three *E. ruminantium pCS20* sequences isolated in this study (MG544303 – MG544305) shared a nucleotide identity of 99.64% and had 100% sequence identity with other GenBank sequences from Ethiopia (GU797236), Benin (KX356089), the Ivory Coast (KX356090) and Cameroon (JQ039914 and JQ039939). As for uncharacterised *Anaplasma* spp. *16S rRNA*, the three sequences from this study (MG637125–MG637127) shared 100% nucleotide identity and showed 100% nucleotide identity with sequences from Japan (AY969011) and the United States (KJ942183).

### Phylogenetic analysis

**FIGURE 2:**
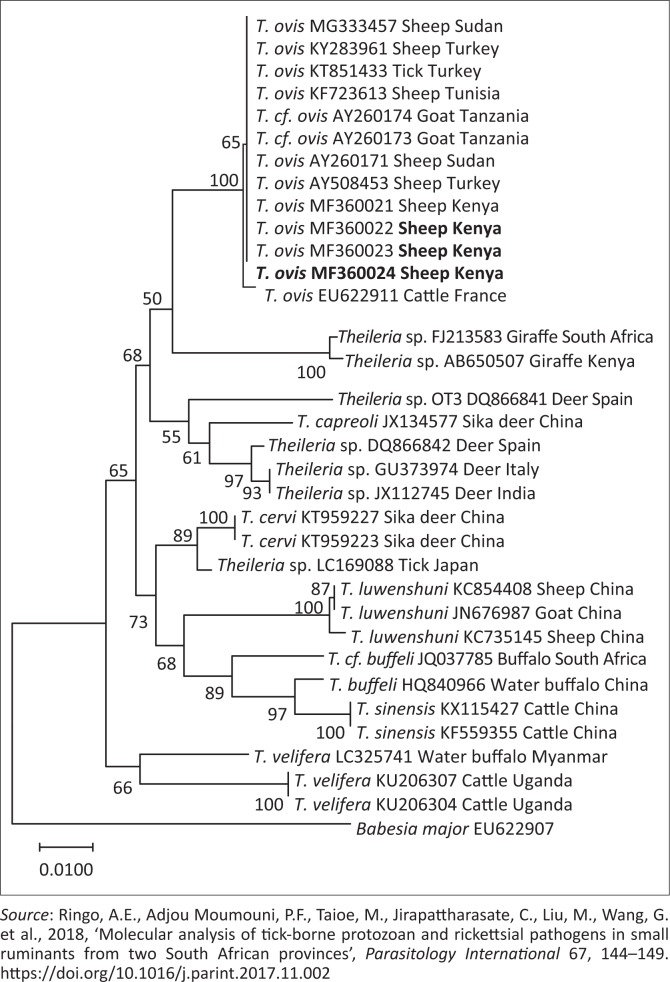
Phylogenetic analysis of *Theileria ovis* based on *18S rRNA*. The sequences generated in this study are shown in bold font. Numbers at the nodes represent the percentage of occurrence of clades in 1000 bootstrap replication of the taxa. The *18S rRNA* gene sequence of *Babesia major* (EU622907) was used as an outgroup.

**FIGURE 3:**
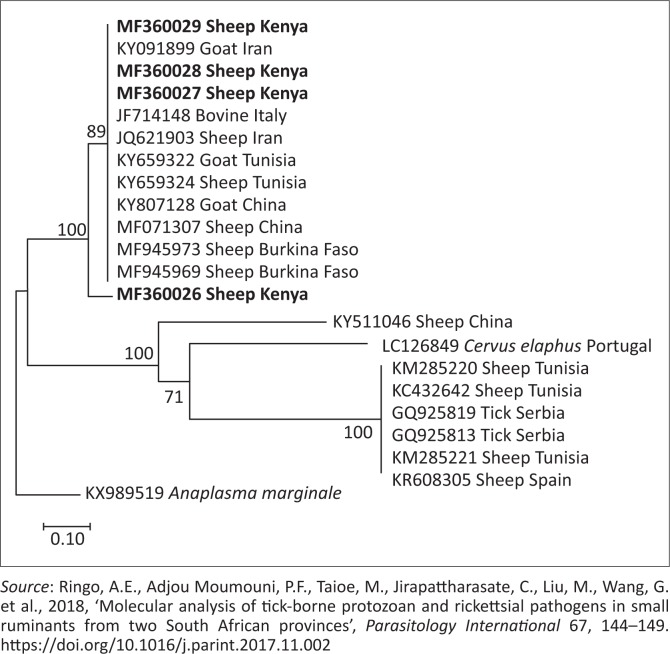
Phylogenetic analysis of *Anaplasma ovis* based on *AoMSP4*. The sequences determined in this study are shown in bold font. Numbers at the nodes represent the percentage of occurrence of clades in 1000 bootstrap replication of the taxa. The *MSP4* gene sequence of *Anaplasma marginale* (KX989519) was used as an outgroup.

**FIGURE 4:**
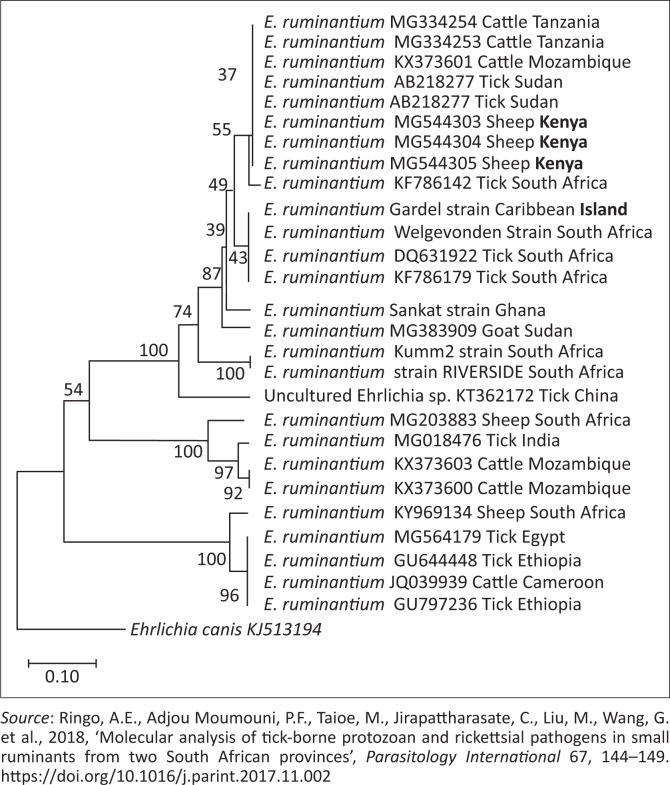
Phylogenetic analysis of *Ehrlichia ruminantium* based on *pCS20*. The sequences in bold font are from this study. The numbers at the nodes represent the percentage of occurrence of clades in 1000 bootstrap replication of the taxa. The *pCS20* gene sequence of *Ehrlichia chaffeensis* (CP007477) was used as an outgroup.

**FIGURE 5:**
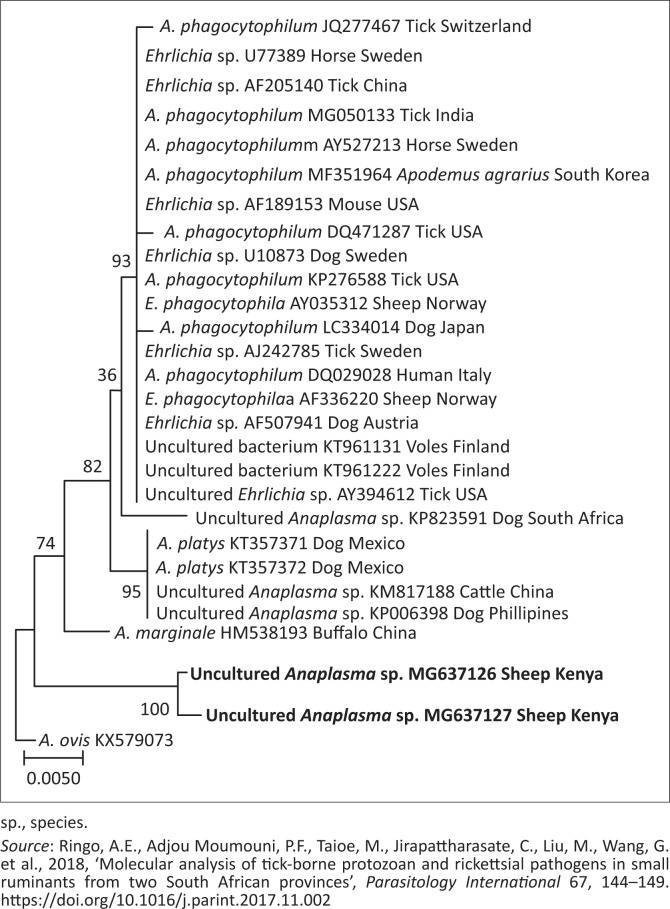
Phylogenetic analysis of uncharacterised *Anaplasma* spp. based on *16S rRNA*. The sequences in bold font are from this study. The numbers at the nodes represent the percentage of occurrence of the clades in 1000 bootstrap replication of the taxa. The *16S rRNA* gene sequence of *Anaplasma ovis* (KX579073) was used as an outgroup.

Phylogenetic trees of *T. ovis, A. ovis*, uncharacterised *Anaplasma* spp. and *E. ruminantium* were constructed based on *18S rRNA, MSP4, 16S rRNA* and *pCS20* gene sequences, respectively, which have been generated in this study and those downloaded from GenBank. The *T. ovis* sequences from this study clustered with the *T. ovis* sequences from the GenBank, making a separate clade from other *Theileria* species (Figure 2). Three *A. ovis MSP-4* sequences from this study were in the same clade, while one sequence formed a separate branch (Figure 3). Similarly, for *E. ruminantium*, all the three sequences from this study were located in the same clade (Figure 4). Meanwhile, the uncharacterised *Anaplasma* spp. sequences of this study appeared in the same clade separated from other *Anaplasma* sequences from the GenBank (Figure 5).

## Discussion

Despite the wide distribution of ovine TBDs in tropical and subtropical regions of the world (Ros-García et al. 2013), and how important they are in livestock improvement (Bilgic et al. 2017; Jongejan & Uilenberg 1994; Jonsson et al. 2008), very little information is available regarding their presence and distribution (Ros-García et al. 2013). In the present study, we performed molecular detection and analysis of tick-transmitted protozoan and rickettsial pathogens in blood samples of sheep collected from Homa Bay and Machakos counties, Kenya. The following pathogens were detected: *T. ovis, A. ovis, E. ruminantium* and uncharacterised *Anaplasma* spp.

Theileriosis in sheep can be caused by several *Theileria* spp. (Berggoetz et al. 2014) – in this study, only *T. ovis* was detected (51.3%). The study revealed *T. ovis* as the most prevalent pathogen, which is in contrast to previous studies (Mwamuye et al. 2017; Omondi et al. 2017; Wamuyu et al. 2015) that did not detect this pathogen in Kenya. However, Adjou Moumouni et al. (2015) reported this pathogen in cattle reared in the town of Ngong which is in Kajiado, a county bordering Machakos county in the south-west. The prevalence of *T. ovis* was compared to other studies from neighbouring countries. In Ethiopia, Gebrekidan et al. (2014) reported a prevalence of 92%, while El Imam et al. (2016) reported a prevalence of 88.6% in the Sudan. Normally, *T. ovis* is known to cause a benign type of theileriosis and is less pathogenic to sheep (Razmi et al. 2003; Schnittger et al. 2000; Uilenberg 1995). This protozoa is considered of low economic importance in sheep (Mtshali et al. 2016). However, it cannot be neglected, as it can cause disease in sheep under stressful situations.

The *18S rRNA* gene sequence of *T. ovis* from this study shared high identity (100%) with isolates from neighbouring countries including the Sudan (AY260171 and MG333457) and Tanzania (AY260174). This could be because of the movement of animals in huge numbers in this region by the pastoralists, which leads to an increased probability of animals from different areas crossing over in search of grazing pastures. This could lead to transmission of ticks from one herd to the other. Moreover, the phylogenetic tree showed all *18S rRNA* sequences (Figure 2) in this study in the same cluster with other *T. ovis* sequences extracted from the GenBank, which suggest that similar genotypes are circulating in the field.

Anaplasmosis caused by *A. ovis* generally is considered to cause sub-clinical symptoms in sheep, although this disease has been reported to be prevalent in high severity in Bighorn sheep (Renneker et al. 2013; Tibbitts et al. 1992). Acute disease tends to be associated with stress factors such as co-infection, hot weather, deworming, vaccination, heavy tick burden, long distance transportation and animal movement (Renneker et al. 2013). In this study, a prevalence of 34.2% was detected for this pathogen – this is higher than previously reported in Kenya by Omondi et al. (2017). However, this pathogen was not detected in the Maasai Mara and Shimba Hills National Reserves (Mwamuye et al. 2017; Wamuyu et al. 2015) located in the south-west of Kenya about 150 km from the study areas. Data from other studies in Africa showed a high prevalence of *A. ovis* in Tunisia (Belkahia et al. 2014), Algeria (Aouadi 2017) and South Africa (Ringo et al. 2018). These observations suggest that *A. ovis* has a wide distribution in different geographical areas of Africa. However, in Kenya and other parts of sub-Saharan Africa, large numbers of sheep are owned by the pastoralists, who travel long distances daily in sunny weather, searching for pastures and water (Byaruhanga et al. 2016). This potentially leads to animal stress and eventually may result in severe disease. The low production of local breeds of sheep owned by pastoralists could be associated with this infection as they are easily exposed to stress and to pathogens that are endemic in sub-Saharan Africa (Bilgic et al. 2017).

Phylogenetic analysis of *A. ovis MSP4* revealed that the three sequences isolated in this study appear in the same clade with other sequences extracted from the GenBank (Figure 3), while the remaining sequence from this study formed a separate branch (Figure 3). This shows considerable genetic divergence of sequences isolated from this study, revealing that different genotypes of this parasite are circulating in the field.

*Ehrlichia ruminantium*, transmitted by ticks of the genus *Amblyomma*, causes heartwater in domestic and wild ruminants in sub-Saharan Africa, Madagascar and some Caribbean islands (Allsopp 2010). The disease is one of the major obstacles to improving livestock production in Africa (Cangi et al. 2017). In this study, *E. ruminantium* was detected in sheep from both Machakos and Homa Bay counties, with an overall occurrence of 7.9% – this supports the previous studies in Kenya (Omondi et al. 2017) that reported a similar prevalence. The presence of several *Amblyomma* tick species in Kenya, including *Amblyomma variegatum*, has been reported by Omondi et al. (2017), which suggests that ovine ehrlichiosis is endemic in Kenya. In the phylogenetic analysis, all the three sequences obtained in this study (Figure 4) clustered together, which shows that the gene is highly conserved in the study areas.

The uncharacterised *Anaplasma* sp. was detected in this study. This study reports the pathogen for the first time in Kenya. The overall prevalence is 31.6% from both sampled counties. Phylogenetic analyses showed the two sequences of this bacterium clustered together (Figure 5).

The overall co-infection rate in this study was 61.8%, with *T. ovis* showing high association in co-infection. The co-infection (*T. ovis* + *A. ovis*) (27.3%) was the most common and this could be associated with a relatively higher infection rate of the two pathogens in this study. Moreover, the two pathogens share the same vector, *Rhipicephalus evertsi evertsi*, which has been reported in Kenya by Omondi et al. (2017). In the two counties, the co-infections were relatively similar, except for *T. ovis* + *A. ovis* co-infection which had a prevalence of 4% and 14% in Homa Bay and Machakos, respectively. This could be attributed to the fact that the two pathogens had higher infection rates in both counties and Machakos had a higher number of samples compared to Homa Bay.

In this study, all pathogens were detected from apparently healthy animals, which suggest that sheep in Kenya have established an enzootic stability status against these pathogens. It shows that animals were infected at the early stages of their lives and developed immunity against the detected pathogens. These animals carry the pathogens without displaying any clinical signs, but they continuously transmit the pathogens to ticks and eventually to new animals.

*Babesia ovis* and *Theileria lestoquardi* were not detected in this study, which supports the previous study (Omondi et al. 2017), although further studies with greater coverage may be needed to ascertain their absence.

## Conclusion

The results obtained in this study indicate the occurrence and diversity of *T. ovis, A. ovis, E. ruminantium* and uncharacterised *Anaplasma* spp. in sheep from Machakos and Homa Bay counties. Mixed infections are common in the study area and, therefore, disease diagnosis can be complex. Consequently, proper diagnostic tests are required for accurate diagnosis. Further studies covering larger sample size and wider geographical coverage, including blood work, more epidemiological data and tick collection are required to estimate the risk factors associated with these diseases and their economic importance.
